# “Hello… I'm Here!” A Co‐Productive Qualitative Study Involving Older People With Vision Impairment and Their Experiences of Acute Hospital Care

**DOI:** 10.1111/jan.16648

**Published:** 2024-12-06

**Authors:** Fiona Wilson, Gemma Arblaster, Holly Geraghty, Sydney Graveling, Zahra Hussain, Nicola Jackson, Zaina Qamar, Elliot Rook, Elena Starsong

**Affiliations:** ^1^ School of Allied Health Professions, Nursing & Midwifery The University of Sheffield Sheffield UK

**Keywords:** acute care, older people, participatory research, sight impairment, sight loss, vision impairment

## Abstract

**Aim:**

To understand the experience and care needs of older people living with vision impairment in the acute hospital setting.

**Design:**

A qualitative study using co‐productive user‐based design.

**Methods:**

Seven older people living with vision impairment and six healthcare students collaborated in a series of six researcher facilitated co‐productive workshops. Recorded data were analysed using thematic analysis.

**Results:**

The needs of older people with vision impairment are often overlooked in the acute hospital setting. Four themes identified (1) Trauma and Loss; (2) Vulnerability and Feeling Invisible; (3) Being disabled, and (4) Feeling safe. Trauma associated with vision loss was acute, particularly if acquired during the hospital admission. The experience of vision impairment coupled with illness served to heighten feelings of vulnerability and needs were often overlooked creating a sense of invisibility. The hospital environment was experienced as disabling, and care needs were not always acknowledged. Supportive communications and access to own audio/supportive devices were vital in supporting a sense of safety.

**Conclusions:**

Our study is significant in highlighting the increasing global importance of vision aware care in the context of an ageing population. The study is also unique in illustrating the potential for inclusive and creative co‐productive approaches which engage both older people with sight loss and healthcare students to promote vision aware practice. Supporting vision impaired older people through diagnosis, care and discharge is vital for promoting equitable positive health outcomes and quality of life.

**Impact Statement:**

*What problem did the study address?* Despite a high prevalence of sight loss within the older patient population, it is unclear how the acute hospital setting supports people living with vision impairment.

*What were the main findings?* The hospital environment and lack of vision aware care can impact negatively on the care experience resulting in poorer physical and psychosocial wellbeing.

*Where and whom will the research have an impact?* This work will inform the development of vision aware care educational resources, policy, and practice.

**Implications for the Profession and/or Patient Care:**

Vision aware care is vital for supporting quality of life and health outcomes for all older people.

**Reporting Method:**

EQUATOR guidelines have been adhered to using the COREQ checklist.

**Patient or Public Contribution:**

The charitable organisation Sheffield Royal Society of Blind (SRSB) was involved in the planning of the research to ensure that volunteer participants could take part and the conduct of the research was inclusive to their needs.

**Summary**

- What does this paper contribute to the wider global clinical community?
  ○ Global sight loss is increasingly prevalent in the older population but is largely unacknowledged in non‐orthoptic healthcare settings with negative implications for care giving, quality of life, and older people's wellbeing.
  ○ Working with vision impaired people in developing co‐productive research methods can promote novel approaches to sharing their stories in ways which are impactful, inclusive, and empowering.
  ○ Involving healthcare students including nursing students in real world research offers valuable learning opportunities for evidence‐based practice and development of research skills.

## 1 Introduction

Many older people accessing acute hospital care are living with several co‐ morbidities and disability, including vision impairment. The World Health Organization (2019) estimates that globally there are 2.2 billion people living with vision impairment, and whilst all age groups are affected, sight loss is described as one of the most common disabling conditions in the aging population (Burton, Gibson, and Shaw 2016) and is a growing global public health concern (Swenor and Ehrlich 2021). In the United Kingdom (UK), it is estimated that over 2 million people are living with sight loss, and 80% of these are 65 years and older (Royal National Institute for the Blind 2021). Common causes of vision loss include cataract, age related macular degeneration, glaucoma, and diabetic retinopathy. Stroke can also lead to vision impairment in approximately 60% of stroke survivors (Rowe et al. 2022). Currently, two‐thirds of acute hospital admissions in the UK involve individuals aged 65 years or older, and this figure is expected to rise in light of the aging population (British Geriatrics Society 2023). Many patients are therefore likely to be living with vision impairment, but it is not clear how vision impairment impacts on the acute care experience, or what adaptations are required to support person centred vision care. This paper describes a co‐productive study involving both older people with vision impairment as participants and healthcare students as co‐researchers, to explore the experience of acute hospital care for older adults with vision impairment.

## 2 Background

Vision impairment is a term broadly used to describe when a person experiences an impairment of their vision. This can include being certified as severely sight impaired (previously categorised as blind), certified as sight impaired (previously categorised as partially sighted) or being described as having low vision, typically when vision is reduced compared to normal levels but does not meet the criteria for certification. Living with vision impairment is particularly challenging for older people. World Health Organization (2019) asserts that in older adults, vision impairment contributes to greater social isolation, reduced mobility, and higher risk of falls and fractures, with many having a greater need for nursing or care home support.

The incidence of vision impairment in the acute hospital setting is unclear. Rowe et al. (2022) identified that 58% of people who were admitted to a stroke unit had vision impairment, rising to 73% in stroke survivors. One in four older people in the UK aged 80–85 years required a vision outpatient appointment in 2019/202 (Public Health England 2021). Such figures suggest that vision impairment is highly prevalent in hospital patients aged over 65 years, but exact numbers are unavailable and likely underestimateneeds. In the context of a globally aging global population, vision impairment presents as a worldwide public health issue with implications for health care usage, productivity, and quality of life (World Health Organization 2019) and requires consideration of future care provision. Currently, people who are severely sight impaired are at greater risk of hospital admission and falls and have more and longer periods of inpatient hospital care (Evans, Smeeth, and Fletcher 2008; Brundle et al. 2015) with a delayed return to independent living, higher rehabilitation reablement needs (Rowe et al. 2016) and higher mental health needs (Demmin and Silverstein 2020). There is also evidence that older people with vision impairment and dementia face particular challenges in terms of potential distress and trauma (Watkinson and Blood 2014).

Despite the potential impact of sight loss on care and health outcomes, (Watkinson and Blood 2014) contend that health professionals do not always acknowledge the significance of vision impairment for individuals with negative consequences for quality of care and referral to appropriate services. Carpenter et al. (2020) and Leroi et al. (2022) assert that sensory loss, including hearing and vision impact negatively on patient and health professional communications resulting in suboptimal assessment and care in a range of contexts including dementia and palliative care. Lack of appropriate assessment of needs or professional awareness also results in fewer appropriate referrals to vision support services (Stolwijk et al. 2023). Vision impairment, along with the support and treatment associated with it, varies significantly among different groups of older individuals (Royal National Institute for the Blind 2021). In the UK, there is a notable inverse relationship between social positioning and vision health within the older population (Whillans, Nazroo, and Matthews 2016). Inequalities in vision health therefore has implications for health care provision, public health, and policy interventions.

The UK Equality Act 2010 (GOV.UK 2010) supports an agenda of equality, diversity, and inclusion (EDI) ; however, vision impairment appears to be largely ‘invisible’ in non‐ophthalmic health care settings. The Sunflower Scheme (Hidden Disabilities Sunflower Scheme 2024) promotes wider recognition of invisible disabilities, including sight loss, in public places, including healthcare. However, despite the existence of guidelines to support people with vision impairment in hospital settings (e.g., NHS Greater Glasgow and Clyde 2019), we were aware both as clinicians and as individual and family users of hospital services that negotiating hospital care with vision impairment can be challenging. Similar experiences are reported by the Royal National Institute for the Blind (2021). Given the prevalence and impact of vision impairment in older people, this study therefore aims to explore how acute hospital settings can adapt and support older people living with vision impairment.

## 3 The Study

This study adopted a participative and co‐productive model based on user‐centred design principles (Bate and Robert 2007). Participatory research practices are situated within models of social justice and inclusivity (Dale 2010) and opportunities to explore and co‐produce aim to ensure a ‘nothing about us without us’ approach, particularly in the field of disability (Rix et al. 2020). Concerns have been raised regarding the extent to which participatory research practices can effectively challenge existing research hierarchies and promote empowerment and inclusivity (Walmsley, Strnadová, and Johnson 2018). However, the Royal National Institute for the Blind (2015) emphasizes the importance of actively engaging individuals with vision impairment in the research process to ensure that services and policies are both relevant and accessible.

Participatory research methods are commonly based on visual imagery (Rix et al. 2020) employing strategies such as photovoice (Richards et al. 2023) and pictor (Hardy, King, and Firth 2012) which enable people to ‘show’ their stories through visual representation. Despite the availability of visual aids and supportive technologies, visual approaches were likely to be challenging in the context of vision impairment. Duckett and Pratt (2007) emphasize an approach of partnership working and inclusivity when working with people with vision impairment, usually through one‐to‐one interviews and focus groups. More creative approaches to engagement were less evident in the literature. Our study was committed to supporting modes of co‐production which did not rely on visual prompts or semi‐structured interviews and focus groups but would instead enable participants to ‘show’ their experiences in ways which were inclusive, co‐productive, and innovative.

An additional unique aspect of our study included the involvement of healthcare students as co‐researchers in the study. The Royal College of Nursing (2024) states that active nursing engagement with research can enable new knowledge, contribute to the evidence base, and transform the patient experience (Royal College of Nursing 2024). As clinical academics (GA: orthoptist lecturer, FW nurse lecturer) with a role in developing evidence‐based practice, we were keen to involve students in a pedagogical approach which positions students as co‐producers of knowledge (Jones et al. 2012); thus, aligning to a ‘student as partner’ model of learning advocated by Neary (2012). Dancis, Coleman, and Ellison (2023) suggest that universities are sites of critical learning and yet rarely invite undergraduates to meaningfully participate as co‐researchers. Although clinical placement and timetabling commitments restricted student involvement, we felt that their involvement in the study would be beneficial, particularly as they are likely to be assisting older individuals with vision impairments in clinical settings. Their participation would contribute to the development of research skills, support placement learning, and facilitate knowledge exchange that could be directly applicable to practice. It should be noted that all were second year students and research methods and methodologies formed part of their on‐campus learning.

We were sensitive to the potential for student/lecturer power dynamics to inhibit a student involvement as co‐ researchers. Students were provided with a paid research contract and could decide hours and ongoing involvement depending on availability and commitment. We found that as the project developed, so did the student role in which they moved from novice to a skilled role as facilitators and co‐producers. We ourselves gained from the student perspective in terms of their current practice and developing relationships with participants. This study therefore supported creative and inclusive approaches to researching with vision impaired older people, as well as providing opportunities for healthcare students to gain ‘real world research’ experience and provide first hand insights into vision impairment and care needs.

### 3.1 Aims and Objectives

This study offers an original and unique approach to exploring the needs of vision impaired older people in health care research. The primary aim explored the experience of acute hospital care for older patients with sight loss. Three objectives were identified, first to explore the experiences of older people with vision impairment of acute hospital care to identify good practice in supporting care in the acute hospital setting; second, to contribute to creative and inclusive participatory research approaches with vision impaired older people and third, to explore the potential for students as co‐producers of research.

## 4 Methods and Methodology

### 4.1 Design

We adopted a participatory co‐productive qualitative approach, working with older people with vision impairment and a small number of health care students, to reflect upon and explore people's experience. Thematic analysis (Clarke and Braun 2017; Nowell et al. 2017) was used to explore qualitative data and support a reflexive approach to listening to experiences, inductively identifying patterns within first line coding to develop themes.

### 4.2 Theoretical Framework

Our approach adopted a social justice model of user‐based health design utilising a co‐productive approach to engaging with others as equal partners (Bate and Robert 2007; Fisher, Craig, and Chamberlain 2019). Such an approach aims to identify what is most relevant to participants, identify issues that may be less evident to researchers, and improve the experiences of care (Borgstrom and Barclay 2019) which is particularly salient for populations whose voices can be marginalised within mainstream healthcare.

### 4.3 Study Setting and Recruitment

A local charity organisation, Sheffield Royal Society for the Blind (SRSB), offering support to people living with vision impairment had strong links to GA and her role within the department of ophthalmology and orthoptics. Both GA/FW were experienced clinicians and health researchers and SRSB were keen to support the study. A series of six workshops were held on site at the SRSB. The location was able to provide hospitality, counselling support, and key workers and was well known to participants as a weekly meeting space. Support workers were also present within the workshops and available for counselling support.

Recruitment was purposive and facilitated by the SRSB via a study invitation through digital social media and newsletters and invited participants aged over 60 years with recent (in the last year) patient experience of acute hospital care. The organisation acted as a gatekeeper, and their ethos was as an enabler in supporting clients and advocacy work.

An email was also sent to all second‐year students in the School of Allied Health Professions and Nursing & Midwifery (AHPNM) inviting interest in participating in the study. All vision impaired participants were offered payment vouchers in line with a participatory research ethos as outlined by the National Institute for Health Research (2019) and Rutter et al. (2024), along with hospitality and travel expenses. All students were offered a casual worker contract with flexibility around study and placement commitments. At the close of the study, a postgraduate student, from ophthalmology and orthoptics, joined to facilitate the production of the final co‐produced learning outputs.

Consideration was given to study information and consent in the context of vision impairment. The study team was able to attend a scheduled social activity day to introduce ourselves in person, describe the study, and respond to any queries. Consent forms were completed with the assistance of the link workers. Only one participant used braille but was verbally able to consent via a link worker. We also utilised the first meeting (labelled workshop 1) to repeat study information, describe co‐production, and explore participation and how participants might wish to contribute. Ongoing consent was renegotiated at each of the workshops throughout the study.

Seven older people aged between 62 and 83 years (four women, and three men) participated in the study. Eight participants agreed to take part in the study but one withdrew due to ill health. Two potential participants were excluded as under 60 years of age.

### 4.4 Inclusion and Exclusion Criteria

Older people aged over 60 years with recent experience of acute hospital in patient care and with a range of vision impairment were approached via the organisation's communications/newsletters.

Participants presented with a range of co‐morbidities including for example diabetes, stroke, and hearing impairment. Whilst co‐morbidities impact on the experience of hospital care, these did not inform inclusion/exclusion criteria as the focus was primarily on the impact of vision on the hospital experience. It should be noted that those participating did not indicate hearing impairment. Eight participants agreed to take part in the study but one withdrew due to ill health.

### 4.5 Data Collection

A series of six workshops were facilitated by GA/FW as workshop leads, with students supporting individual tables as part of a small group work format. Workshops lasted between 60 and 75 min, and all workshops were audio recorded and transcribed verbatim. After each workshop, staff and students had a debrief meeting and immediate reflections were recorded. Later reflections were encouraged and documented (see Table 1 for an overview of workshops).

**TABLE 1:** Overview of workshops.

Workshop	Activity
Workshop 1	Introduction, information, consent
Workshop 2	Testing different activities
Workshop 3	Exploration of experiences
Workshop 4	Exploring themes
Workshop 5	Developing a learning resource
Workshop 6	Reviewing resources and next steps

The first workshop (introduction, information, and consent) introduced the study. We provided examples of co‐productive participatory studies, outlined aims, and invited participation, consent, and feedback. Participants indicated an altruistic desire to support the study and inform care‐giving practices in the acute hospital setting.

In workshop two, (testing different activities), participants worked in small groups of two to three, around smaller tables, spread across two rooms. The research team including students acted as facilitators and participants explored a range of different approaches to support vision impaired people in sharing their experiences. It was envisaged that tactile objects could aid discussion and support focus. Object elicitation in which a range of objects are utilised to prompt discussion has been used in user‐based design to explore reflections of a phenomenon (Fisher, Craig, and Chamberlain 2019) but not specifically within the context of vision impairment. The proposed activities therefore utilised an object elicitation approach to facilitate discussion around ‘emotional touchpoints’ in which tactile prompts could stimulate recall of emotional (low or high) responses to the hospital journey (Bate and Robert 2007; Borgstrom and Barclay 2019). Interventions for object elicitation were devised through discussion and reflection as a team. Participants were also asked to consider recording their experiences either alone or as a diary format or with a researcher in one‐to one interviews. They were also invited to consider music or recordings of poems or personal podcasts to reflect upon experiences. At the close of the workshop, participants provided feedback on what they found most engaging and stimulating or suggested alternative options. Table 2 provides an overview of activities tested.

**TABLE 2:** Workshop two: Testing different activities.

*Activity 1: The Patient Day*—using shaped Post‐it thumbs up/thumbs down to indicate positive and negative aspects of the hospital day routine
*Activity 2: Emotional touchpoints*—using a range of tactile prompts to elicit ‘bumps’ and enablers in hospital admission. Tactile prompts included toy trains and train tracks (flat and raised bridges), tactile bump‐on stickers, tray with raised discs and trains alongside verbal prompts including, ‘What do you hear, remember, feel during the journey’?, What were the ‘bumps in the road’ and the ‘easy parts of the journey’ of your hospital experience?
*Activity 3: Telling through the senses*—can you describe the sounds, smells, touch and sights that remind you of your hospital stay? What made you feel comfortable? What made you feel uncertain?
*Activity 4: Talking about the sounds that remind you of your hospital stay and talking about what these sounds mean to you?* What audio recordings would capture your experiences? Would music capture your experience? What about writing and recording a poem? How important was audio/music/recordings/spoken word to you?
*Activity 5: Would you prefer to explore your experiences in personal recordings or one‐ to‐one interviews?*
*Activity 6. Summary Feedback: Which activities worked?* Discussion space for participants to add and contribute their own ideas for data collection methods as well as provide feedback on which of the above activities were most engaging and stimulating

Feedback from workshop two indicated that some sensory prompts were confusing if not clearly related to the topic (e.g., loose beads, counters). Although there was some interest in recording their thoughts either independently or with a researcher in one‐to‐one interviews, all declined, explaining ‘they did not want to go there alone’ but instead indicated they enjoyed small groups. Participants indicated that the tactility of coloured and shaped Post‐it notes helped retain focus. By working with a student facilitator, it was possible to record verbal points on the note, which could then be posted onto a chart as data. This feedback informed data collection in the subsequent workshop three.

Workshop three (exploration of experiences) had different shaped coloured post‐it notes, and a series of prompts were devised from the feedback from workshop two. See Table 3 for ‘prompts for elicitation’. Small groups, working together with a research facilitator, recorded key words or phrases as dictated onto Post‐it notes. These were then set to card as data. As per all the workshops, discussions were audio recorded and transcribed. Facilitators were invited to record reflections of the workshop.

**TABLE 3:** Prompts for elicitation.

*Activity 1: Thumbs up/down Post‐it notes*: The Patient Day—thinking about a typical day in the hospital—morning/washing and getting ready, lunchtimes and food, afternoon‐drug rounds, investigations, nighttime—sleep and darkness What made you feel comfortable (thumbs up?) What made you feel uncertain (thumbs down?)
*Activity 2: Using the speech bubble Post‐it notes*: Things you have been told or would like to say to others. Can you tell us some positive things that people said to you or that you or did not understand (for example, not introducing themselves clearly?) What would you like to say to other people about your experiences or care?
*Activity 3: Using butterfly shaped Post‐it notes: Raising awareness*: If we could send butterfly notes to stick around the hospital to raise awareness about how to support people with vision impairment—what would you say? What advice would you give to others? What one or two things would you write on your butterfly note?
*Activity 5: Heart shaped Post‐it notes*: “I'm trying to tell you”‐ can you think of something good about your care that made a positive difference that you think other people should know? What were the little and the big things that made a difference to you? What were the positives? Trying to help others understand the problem and give them a solution

Preliminary analysis was presented in workshop four (exploring themes). The transcribed data from group discussions and key words or comments on the Post‐it charts were analysed using NVIVO Plus (Lumivero 2023). This preliminary analysis involved a process of first level coding, identifying patterns, and an inductive exploration of overarching themes as indicated in thematic analysis (Clarke and Braun 2017). GA and FW independently explored codes and worked together to organise as four key themes salient to the experiences of participants. Table 4 provides an example of coding.

**TABLE 4:** Example of coding and thematic analysis.

Code	Text	Developing theme
Unable to reach Ill Nobody had been to me Soiled and wet Unable to reach bell Shouting for ages Eventually The night	I couldn't reach the call bell. When I was very ill, this particular day nobody had been to me, and my bag had got that full, it had started peeling off my tummy. The bed were wet through and I called, I couldn't find the bell because it had fallen side of the bed, and I'm shouting for ages, and eventually someone came to me, but it were the middle of the night. (Calling for help, call bell fall on floor?)	Being dis‐abled Feeling alone/vulnerable Being disabled Feeling alone/vulnerable Feeling alone/vulnerable Trauma

These preliminary themes were used to develop three composite case studies which were presented as audio‐vignettes in workshop four. During the workshop, participants listened intently to assess preliminary themes for salience and resonance. Workshop four therefore presented not only as a form of member validation but also as an opportunity to comment and refine the final themes. Participants indicated the themes resonated strongly with their experiences and highlighted the vulnerability of their experiences. Definitions of saturation have been contested (Saunders et al. 2017). Working participatively, we utilised saturation to capture both the saliency of themes and assess the need for further data collection. Participants indicated that the preliminary audio themes were salient and resonated emotionally, and the group also indicated that further workshops were not required.

Workshop five (developing a learning resource) focussed on developing the themes to inform and co‐produce ‘top tip’ resources for (1) health professionals in supporting acute care and (2) for people with vision impairment and their family/friends when preparing for an in‐hospital admission.

**FIGURE 1:**
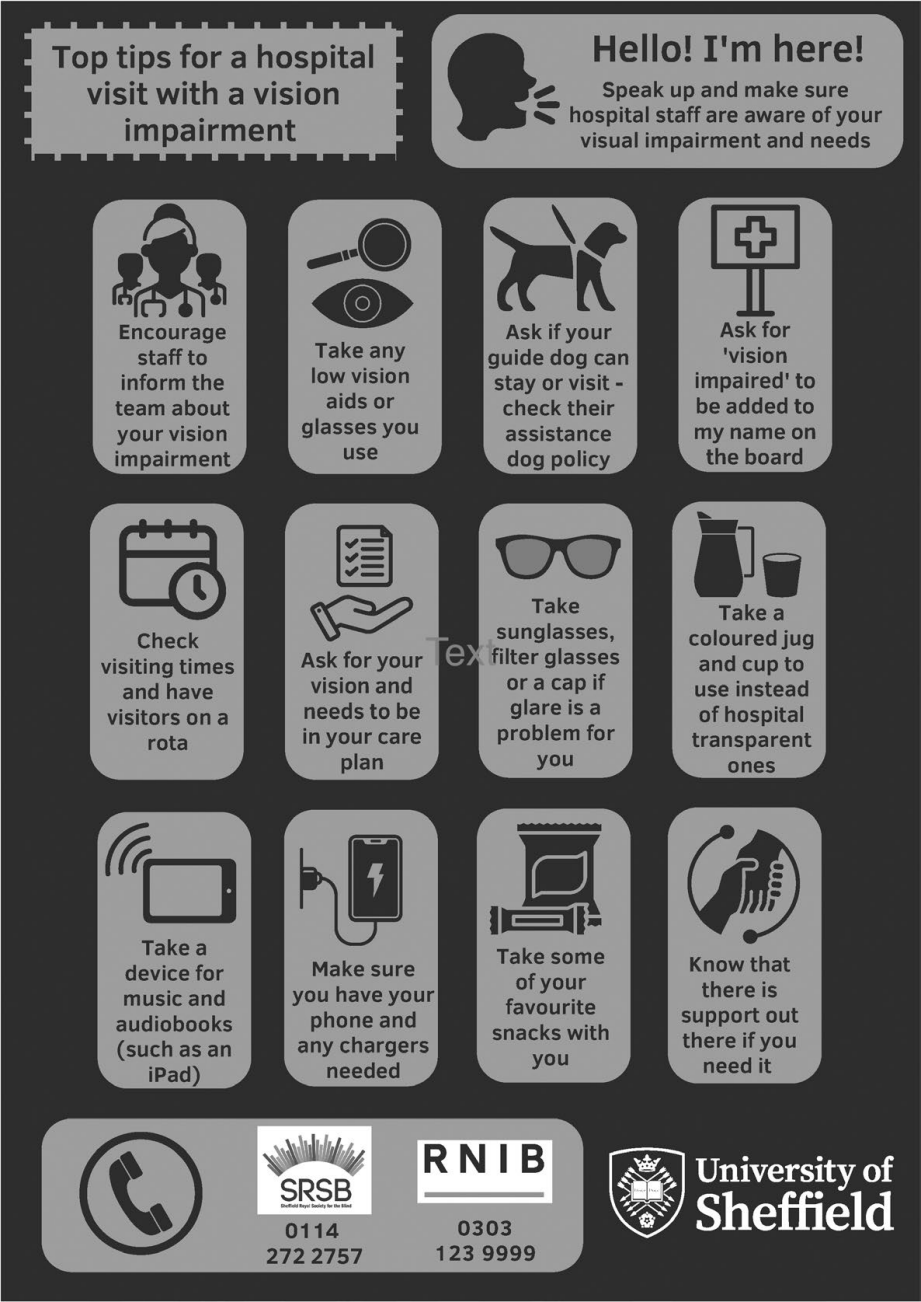
Top tips for a hospital admission.

**FIGURE 2:**
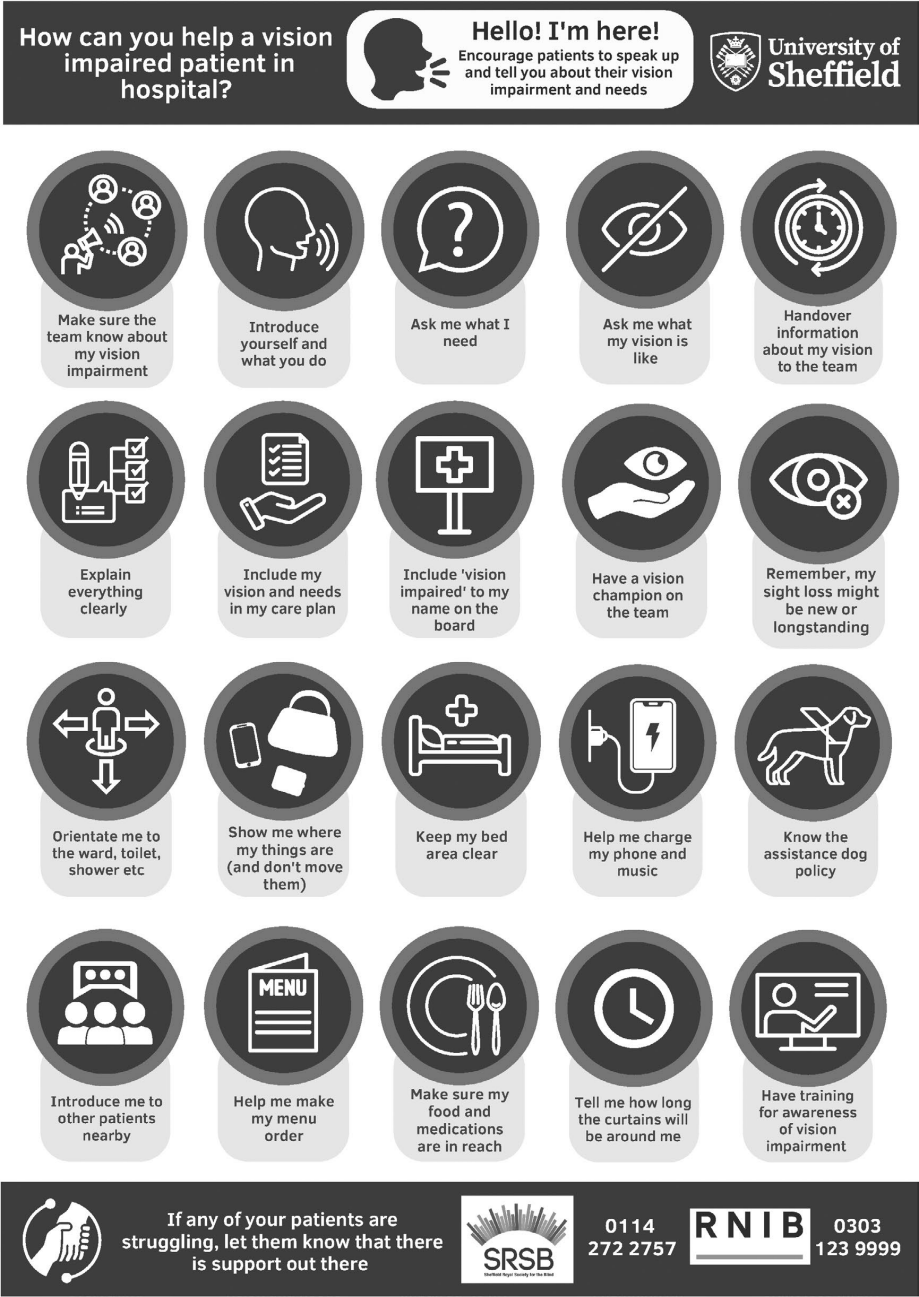
Guidance for staff.

For the final sixth workshop (reviewing resources and next steps), a postgraduate orthoptic student with media skills, finalised two posters based on the ‘Top Tips’ drafted in workshop five, and these were presented for approval. See Figures 1 and 2 for the final output.

Participants were also able to reflect on the study and workshops and indicated that the opportunity to work with students as well as create a tangible output was rewarding. Participants were also keen that their experiences made a difference and were particularly focussed on student simulated learning and raising awareness generally. The sixth workshop also provided a vehicle for study closure.

### 4.6 Data Analysis

As outlined above, the recordings from all workshops were transcribed and checked by the research team, including students, anonymised and pseudonymised, and text entered into the software Quirkos (2023 version Quirkos 2.5.3). This was selected as it offered a visual overview of the data and could potentially enable participants and students to be actively involved in analysis. However, due to time constraints and student placements, the data was recoded by the research team (GA and FW) using NVIVO Plus (Lumivero 2023). The Post‐it notes arranged on card during workshops 3 and 4 were photographed and phrases recorded on them entered as text.

We engaged with participants to explore preliminary findings (see workshop four above). Exploring themes with participants can be described as member validation, enhancing rigour and trustworthiness. However, our approach aligns to that of Birt et al. (2016) in which participant engagement is fundamental to a co‐productive approach in ensuring that participants' own meanings and perspectives are reflected in the analysis. Analysis was undertaken following the principles of thematic analysis (Clarke and Braun 2017; Nowell et al. 2017).

### 4.7 Ethical Considerations

The study received University of Sheffield ethical approval (reference 052639) and all consents and data management met with the UK General Data Protection Regulations Act (GOV.UK 2018) and the research team including students had access to an online secure data site. All stages of the research process were recorded and pseudonymised data archived in a university archive repository.

Rutter et al. (2024) explores the importance of value, trust, and agency in participative research. We were aware of the time, effort, and challenges for participants in contributing to the study and were mindful to ensure that all participants were reimbursed for their involvement. Payments and travel expenses were available to all participants with remuneration also provided to the charity for supporting the work. Exploring what can be difficult experiences has the potential to trigger emotional responses not only in participants but also the research team and the team was sensitive and on‐site support was supported by the charity setting. We were also aware that students may have their own personal experiences and students were invited to reflect on their involvement with opportunities to debrief as a group, and individually, after each session.

### 4.8 Rigour and Reflexivity

The study aimed to engage with all participants equally within an ethos of social justice and attention was focused on transparency, rigour, and reflexivity. The principles of credibility, transferability, dependability, and confirmability in assessing the rigour of qualitative approaches as outlined by (Lincoln, Guba, and Pilotta 1985) and succinctly presented by Nowell et al. (2017). However, we also acknowledge that participatory research requires an additional perspective around rigour and accountability.

Frauenberger et al. (2015) utilise a four‐point framework to consider rigour and accountability in the context of participatory research which is centred around epistemology (e.g., what knowledge is created, how is it shared); values (what values are articulated explicitly and implicitly and the impact of these); stakeholders (who are the key stakeholders and how is the end of project managed), and finally, outcomes (who owns the outcomes, utility of findings and sustainability). The key stakeholders in our study included not only participants but also the wider research team (including students) as well as the SRSB. We engaged with transparent reflexivity throughout, and students (*n* = 6) and the research team (GA/FW) reflected on their own learning as well as power and gender dynamics.

The research team GA/FW were experienced researchers with expertise in co‐productive approaches to research. Initially, we were concerned that undergraduate students would not have the research understanding or training to engage participatively and sensitively in field work. Second year healthcare undergraduate students had undertaken research in practice modules and were familiar with concepts of open questions, confidentiality, and qualitative research generally. The team prepared students prior to each workshop and provided opportunities for debrief and reflection post each workshop. All had clinical practice experience and audio recordings from the student facilitated groups illustrated empathetic rapport with participants, who were in turn keen to support student learning.

We were aware that vision impaired participants may approach the project with prior expectations, and we were keen to be clear about the aims of the study. Member validation can also offer transparency and credibility to an interpretive process and enable participants to comment and control what is produced (Erdmann and Potthoff 2023). Participants expressed satisfaction that their experiences had been listened to and the audio vignettes of case studies were valued. One of the key changes was to recommend the study be retitled ‘Hello! I'm here!’.

## 5 Findings

### 5.1 Participant Characteristics

Eight older people aged between 62 and 83 with varying levels of vision impairment consented to the study but due to illness only seven participated in the workshops (four women and three men). Table 5 provides a participant profile.

**TABLE 5:** Participant profile.

Age (years)	Pseudonym	Vision impairment	Duration of vision impairment	Cause and description of vision impairment
(M/F)				
75 (F)	P01	SI	Acquired, recent	Stroke, R homonymous hemianopia
62 (F)	P02	SI	Acquired, recent	Stroke, R homonymous hemianopia, high myopia
73 (F)	P03	SSI	Acquired, longstanding	Infantile cataract, amblyopia
75 (F)	P04	SSI	Acquired, longstanding and recent	Amblyopia (longstanding) Glaucoma, cataract surgery (recent)
77 (M)	P05	SSI	Acquired, longstanding	Amblyopia, strabismus, age related macular degeneration, diabetic retinopathy, ocular myasthenia gravis
83 (M)	P06	SSI	Acquired, longstanding and recent	Amblyopia (longstanding) Central retinal artery occlusion (recent)
65 (M)	P07	SSI	Acquired, longstanding	Traumatic optic neuropathy

Abbreviations: SI, sight impaired (previously called ‘partially sighted’); SSI, severely sight impaired (previously called ‘blind’), recent ≤ 10 years, longstanding > 10 years.

Six undergraduate healthcare students (four were studying nursing, one orthoptics, and one speech and language) and one postgraduate student (from ophthalmology and orthoptics) participated in the study.

**FIGURE 3:**
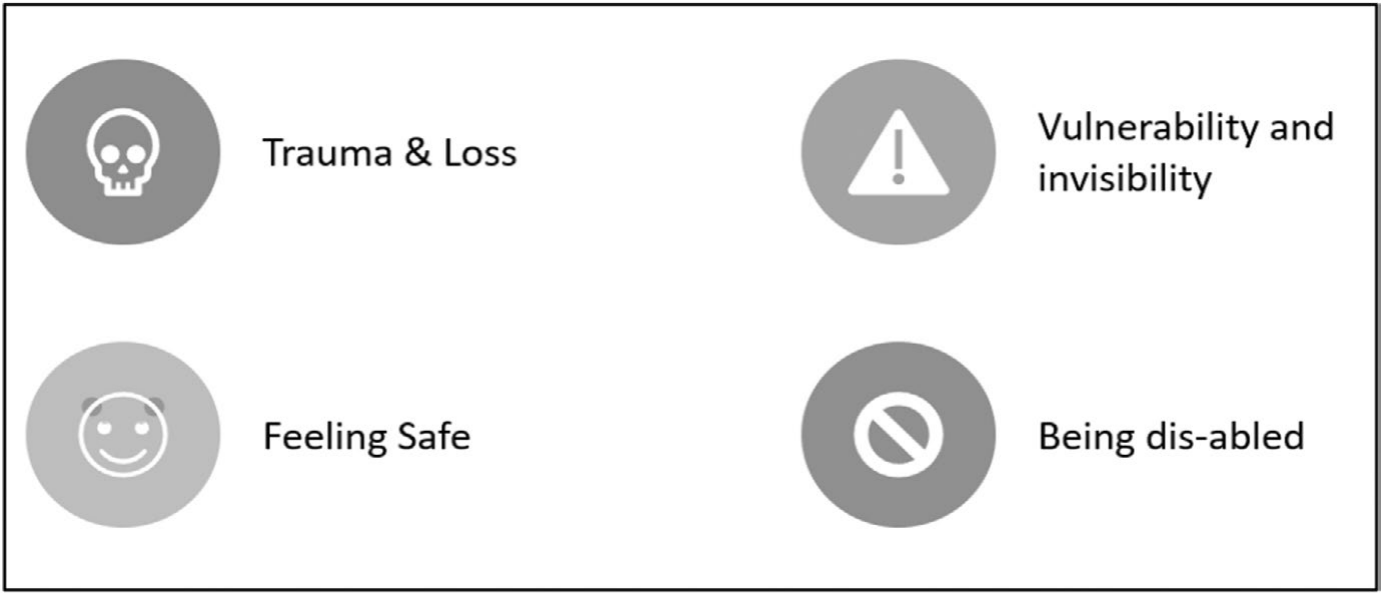
Overview of themes.

Our findings indicate that the needs of vision impaired older people are often overlooked in the acute setting with negative impacts on wellbeing, identity, and dignity. Participants were keen to reiterate differences in their experiences story; nevertheless, despite the uniqueness of each voice, there were similarities captured in the key themes. Thematic analysis identified four themes including: ‘trauma and loss’; ‘vulnerability and feeling invisible’; ‘being disabled’; and ‘feeling safe’. These are presented in Figure 3:

### 5.2 Trauma and Loss

For participants, the impact of sight loss was profound and traumatic. The research team assumed that participants would share experiences of entering an inpatient episode *with* vision impairment, but four participants acquired vision impairment *during* their admission. Their stories therefore triggered high emotions and was impactful learning for all, leading to reflections on whether the trauma of sight loss was always acknowledged in the clinical setting.

I lost my sight about 7 years ago now. It started with glaucoma. Within a few months, they decided to take the cataract off my right eye. I've never had much sight in my left eye anyway from birth. Basically, I went into hospital able to see and woke up blind because it went wrong. From there, really it was quite a shock as you can imagine…. (PO3, F,SI)

I had breast cancer. But it was more traumatic with my eyes than the breast cancer. (PO1, F, S1)

The impact of vision loss was devastating with consequences for quality‐of‐life post discharge which was not always recognised in the clinical setting:

I had 8 years of depression. Really bad depression. Really bad. I didn't want to live. FW Was that due to your eyes?

Yes, you wake up and you, I don't want to get out of bed. (P06, M, SS) (lost sight in hospital)

For one participant there was added trauma of not being able to communicate to her care team that she had lost her sight post stroke. It was not until discharge that her sight loss was apparent to community staff.

When I was in hospital, I had a lot of other things going on, so it took a long time for them to work out what was wrong. I kept saying there was something wrong with my eyes, but my speech was terrible, so I don't think people understood quite what I was trying to say. So, it was a community stroke team that discovered what it actually was, so that's me. (PO2, F, SI)

Not being able to understand why sight loss had occurred was distressing and confusing, and these feelings were amplified if the impact of sight loss was not acknowledged. June was told her sight would return, but this was not the case:

Cos I didn't know what was going to happen, I had no idea, I didn't know whether they kept saying (the nurses) kept saying oh you'll be alright you'll get your sight back, you know they'll sort it. And I didn't get my sight back, you know they lied to me basically. (PO1, F, SI)

In the context of an unfamiliar environment, many described a heightened sensory acuity of non‐visual senses so that conversations and noises were heard acutely. Anticipating the environment through sound was challenging, requiring a constant sense of alert. The following conversation captures the impact of the ‘soundscape’ on the care experience:

The curtains swooshing noise! (PO2, F, SI)

Yeah, those curtains are horrible! (PO3, F, SSI)

Some of them trolleys end up sounding like music. You hear plastic things rattling and you know, water or what's coming? (PO4, F, SSI)

All discussed hearing death and dying as traumatic and distressing, adding to their own sense of loss and distress:

The lady across was dying and I knew she was dying. I used to be a nurse and I knew she was dying. Traumatic, because I knew. I'm rocking now, aren't I? (PO4, F, SSI)

### 5.3 Vulnerability and Feeling Invisible

Trauma and loss contributed to feelings of vulnerability compounded by an unfamiliar environment, illness, and often uncertain health outcomes. Vulnerability was exacerbated in that staff often appeared to be unaware of needs or were even disbelieving: Having to reiterate care needs and explain was not reassuring:

People change shifts and the next lot of people who come in, unless they were told by the other staff that there's a blind man in aisle 6, so he will need a little bit of help getting to and from the bathroom etcetera, etcetera, so if they didn't know, it always goes back to the start. (P07, M, SSI)

Participants also described feeling invisible and abandoned or forgotten such as being left behind curtains. Lack of explanation as to what was happening was disorientating:

You hear noises, but you don't know what those noises are related to. It's explanations of what's happening if you can't see sometimes, or you know, “we are putting these curtains round because we're washing people”, or whatever, “we'll come to you in a minute”, or whatever they're doing, I don't mind what it is, but just let me know, and for how long, as well, as it feels like forever otherwise. (PO1, F, SI)

The following participant was unable to find their call bell and was left for some time resulting in an undignified experience and adding to an overall sense of vulnerability:

This particular day nobody had been to me, and it had got that full, it had started peeling off my tummy. The bed was wet through and I called; I couldn't find the bell because it had fallen down the side of the bed, and I'm shouting for ages, and eventually someone came to me, and I don't know what you call the ladies that do the domestic stuff, but it were the middle of the night. (PO3, F, SSI)

Nearly, all identified the night as the most challenging particularly if there were bed moves in the night or lights turned down. Staff seemed to assume that darkness did not impact on those with vision impairment:

I think it was at night, it was the lack of sound. It was that silence. (P04, F, SSI)

And the darkness and now I sleep with a little night light just because it helps me if I need to get up or and I just I know nobody can see in the dark, but it just really freaks me out a bit still at the moment. (PO2, F, SI)

Interactions underlined the invisibility particularly when interactions were dismissive, and patients were talked over:

One of the nurses came in and said “what do you think she'll want over there for her breakfast” and I didn't know they were talking about me cos I couldn't see you see, I sat there and so this lady who I've got friends with from across (the ward) and she said, “well, don't you think you want to go and ask her”, so I realised that she was talking about me! (PO4, F, SSI—visibly upset)

The sense of invisibility was emphasised by participants who suggested that we retitle our study to reflect their experiences:

I think visual impairment, they don't think you're there. I myself would've put “Hello I'm here”. If you're looking at them and can see they're more liable to say “do you need help?” but because we can't see them, we're not there. (P01, 75, SI)

### 5.4 Being Disabled

A disabling care environment and disempowering care practices were not only undermining but also detrimental to physical and psychosocial wellbeing. Unfamiliar environments or poorly designed ward spaces were hazardous, and independence was undermined by poor care practices or lack of understanding. The design of the environment was disabling:

Yeah, a lot of it and I'd go round to the bottom of the bed and think, well, I knew it was a four‐bedded bay and I was trying to work out how far from the end of the bed and where the gap would be and that's when I've bashed my head and that's how I've ended up with this bruise. (P07,65, SSI)

Everyday design of bed space paraphernalia including jugs, call bells, and tables also impeded independence:

Trying to pour water into a clear glass from a clear jug. You see I love yellow, and why they didn't… if you (use) red or blue, or any colour that is just different than just white. It's like when jugs had the red lids on, and at least you could find the top of a jug… everything, because my call bell, often my call bell was on the floor. Well, I suppose there are signs on the floor and walls but often I cannot see them, even when I can, I cannot read them because they are hard colours to differentiate. (P01, 75, SI)

Not all vision impaired people can access braille but for some the lack of braille was a block to independence:

I know the ward numbers are in print, but it'd be nice to have them numbered in braille as well …. So, if a blind person is, or feels independent like I do they can just get out of bed and they can walk and they can find their own ways to those places rather than having to wait for a nurse to come take them to the loo or the bathroom. (P07, M, SSI)

As well as environmental design, care practices were also disabling. Being moved in the night or transferred to other wards was unhelpful and often required a remapping of the environment. Elements of essential care were overlooked with food, belongings, medications placed out of reach, and minimal support with hygiene as illustrated by the following:

When I was on a ward with 80–90‐year‐olds, I think they thought she's young enough, she can sort herself out. And nobody ever came near me. I asked the other patients where the shower was so I could get a shower on the fourth day, because I hadn't even had a shower or wash. (P01, F, SI)

Access to food was challenging, not simply in terms of where food was placed, but in ensuring orders were processed. This was problematic particularly for those with diabetes:

I didn't get any food cos they'd left the menu on my right side and I hadn't filled it in. I wouldn't have been able to see it anyway to fill it in, but they said oh you didn't fill it in so we assumed you didn't want any food. (P04, M, SSI)

Usual support or coping mechanisms were disrupted by the hospital environment. Cathy relied heavily on her guide dog but this was not supported by the hospital guide dog policy. Visitors brought her dog for 1 h daily contact, causing distress for the dog and herself:

They would let her (guide dog) in for an hour. She was always distressed. They had to drag her out. (P04, 75, SSI)

Fred struggled to maintain his blood sugar levels but was able to draw on his long‐standing relationship with his consultant to regain control of his insulin medication.

So, I tried to explain to the nurses what type of insulin I needed but they wouldn't have it and so I was having hypo after hypo after hypo. I had to ring my diabetes consultant and explain, and they had to ring the ward and said look, he knows what he's doing with his Insulin so please let him have his own pen. (P05, 77, SSI)

Participants cited lack of knowledge around vision impairment as a contributory factor in disabling care practices. Use of canes could signify vision impairment but often participants felt dismissed as ‘not really blind’ which contributed to psychological distress.

I was told by a nurse that I didn't need help, when I was at an ENT specialist—“I've seen you walking, so you don't need any help”. How could someone judge me like that? (P02, F, SI)

Well people make room for me (when using a cane) but then it is a bit embarrassing when I get my phone out. (P06, M, SSI)

Normative understandings of what people with vision impairment can and cannot do were also reflected in patient understandings which were experienced as devaluing:

I heard a woman say, “what do you put her down there for?” I can't see the telly for her and she's not watching it. And I thought ‘you ignorant pig’. (P03, F, SSI)

Some were able to challenge care practices and were assertive. For example, Harry described himself as blind since childhood and was confident in asserting needs.

Another sought her daughters help in mediating support for her needs:

(My) Daughter … said “I'm coming in at 9am with my mother and staying until 8 o'clock”. Because I was 5 days having body washes. I still don't know how you use the showers in the [x] hospital. She came and said “my mother is not being left in hospital in these conditions”. (P03, F, SSI)

Generally, however, participants described care settings as challenging and disorientating. One aptly summed up her hospital experience:

I don't know, but I definitely feel as though I am losing my independence. (P01, F, SI)

### 5.5 Feeling Safe

Despite negative examples given in the previous themes above, there were instances of positive and supportive staff interactions which were considered supportive and promoted feelings of safety and significance. Staff who were considerate or went the ‘extra mile’ were highly valued and practical gestures (such as checking that chargers were available or call bells in reach) contributed to a sense of safety:

Some staff were smashing with me. Like when someone just comes and asks Have you got everything you need? Have you got the buzzer? Do you need to charge your phone? (P04, F, SSI)

In the morning, I had forgotten my charger and they said, “Don't worry, I'll find you one” And the nurse comes back with one and says, “here you are, all set up”. And if I couldn't see what I was doing with my phone sometimes, as he walked past, he used to come and I would say Charlie, and he would say “I'll do it for you, don't worry”. (P07, M, SSI)

And another would come around and say are you warm enough; do you need another blanket. It's just little things. (PO4, F, SSI)

Some wards fostered a sense of a patient community which was important for feeling safe, particularly when fellow patients were supportive and provided practical guidance for navigating spaces and belongings. Being introduced to other patients was therefore important.

That is where the patients on the ward were handy for me. And they would watch me walk down. And they would say ‘now you are at the end bed, move to your right, keep walking, move to the left, keep walking’. That is how I got my way around. (P07, M, SSI)

Where the environment was less supportive, patients coped by creating their own ‘spaces’ for feeling safe. All utilised audio‐visual support to promote a place of refuge. Music and audio books were essential for coping with sensory overload, loneliness, and vulnerability, helping to block out unwanted stimuli and connect with family and friends, particularly during covid restrictions.

My phone was a lifeline to family and friends particularly during covid restrictions. (P05, M, SSI)

One participant even used recordings of her husband to hear a reassuring familiar voice:

I take four or five recordings of my husband, and that's comfort for me. (P04, F, SSI)

It was also a way of maintaining identity and bonding with staff; For example, Frank would share his music with the nurses who couldn't believe he was into heavy metal:

Student: Does that music help you cope?

Without a doubt. Without a doubt. Hundred per cent. It's kept me alive, my music, I don't know what I'd do without it. (P06, M, SSI)

Morning sounds were particularly reassuring and important when contrasted to the challenges of nighttime. The acuity to sound and noise rendered interactions which held an ambience of warmth and ‘normality’ as highly positive and reassuring.

I think that was a thumbs up for me as well. The actual noise in the morning. That silence at night when you couldn't sleep. Not nice. (P02, F, SI)

I always used to listen to the trolly going round in the morning with the fresh water and a pot of tea. (P07, M, SSI)

Oh my gosh, that was a cleaning lady that used to come in in the morning and she used to come in and sing and cheer the day up. (P04, F, SSI)

## 6 Discussion

This study provides a critical insight into the experience of acute hospital care on older people living with vision impairment and is especially significant given an increasingly ageing patient population and associated prevalence of age‐related vision loss (Swenor and Ehrlich 2021). The study had three aims: to understand vision impaired older people's experiences of acute care settings; explore inclusive nonvisual co‐productive approaches to researching with vision impaired people and finally to explore the potential for facilitating student learning around real world research. These will be discussed in turn below.

The impact of vision impairment has implications for physical and mental wellbeing and vision needs and can be framed as a human rights issue (Swenor and Ehrlich 2021). In our study, the experience of participants in terms of quality of life coupled with a lack of health professional awareness around vision health raises questions of equity and equality. Globally, it is recognised that vision health is symptomatic of intersectionality across a range of dimensions in which women, older people, rural populations, and minority ethnic communities are at higher risk of poor vision and are more likely to experience structural barriers in accessing vision care and support (Burton, Gibson, and Shaw 2016; World Health Organization 2019). Health inequalities related to socioeconomic positioning and vision impairment are prevalent within the UK (Sharf et al. 2017) and predicted to continue particularly in the context of ageing minority ethnic communities (Heinze and Jones 2024) with implications for health policy and practice (Whillans, Nazroo, and Matthews 2016). The impact on individuals and quality of life as well as resulting costs to the health service in terms of falls, delayed discharge, and hospital admission is likely to be high. It is clear therefore that eye health is an increasingly pressing issue requiring attention to practice, policy, and practice across the life course.

The emotional impact of vision loss is significant, and, in our study, loss of sight was traumatic and particularly harrowing for those whose sight deteriorated during the hospital admission. Participants described profound distress which extended beyond discharge. The concept of biographical disruption (Bury, Gabe, and Bury 2004) and consequent impact on self, identity, and relationships is salient in the experiences presented. Biographical disruption captures a rupture in internal and external identity for the individual and their families. In the context of such disjuncture, it is remarkable that vision loss or impairment was not always fully recognised or communicated by staff, resulting in reduced options for participants to discuss, understand and make sense, or seek information and prepare for discharge.

Responses by healthcare staff to patients with vision loss suggest at worst a dismissive stigmatising and disablist ‘othering’ or at best, lack of understanding and acknowledgement. Certainly, the experiences recounted in our study reflect a stigmatising sense of abandonment, lack of understanding, and care practices which failed to acknowledge psychosocial or physical needs. Similar findings are reflected in other studies for example, Hanna, Mercer, and Rowe (2020) in the context of stroke suggest that there is a complete lack of visual care, whilst Percival and Hanson (2005), and a recent RNIB report (2024) identify, that despite the devastating impact of vision loss, there is limited recognition of support needs. Lack of awareness around vision conditions and visual impairment may account for staff responses, possibly reflecting an ableist dichotomy of understanding around vision impairment (seeing vs. blind) rather than a range of impairment.

Ableist responses to disability is described through the construct of ‘suspicious bodies’ in which disability (particularly if not easily identifiable) can be regarded as ambiguous and difficult to categorise (García‐Santesmases and Sanmiquel‐Molinero 2022). Stigmatised responses to disability can be experienced as trauma and are described by Watermeyer (2013) as a symbolic violence resulting in stigma, liminality, and abjection. In our study, participants described a sense of invisibility in which they were either disbelieved as ‘not really’ blind, dismissed, or constructed as ‘other’. The concepts of stigma and spoiled identity in the context of vision loss can impact on the self, resulting in a lack of confidence and embarrassment (Hanna, Mercer, and Rowe 2020; Dale 2010). Such emotions were reflected in our study in which the spilling of water was presented as both outrage at the lack of help and embarrassment. Unsurprisingly participants requested a renaming of the study as “Hello, I'm Here!” to reflect their perceived invisibility.

Understanding the needs of older people with vision impairment as multifaceted is an issue of social justice (Dale 2010). Ableist understandings of vision impairment impact negatively on the possibilities for supportive communication, emotional, and practical support which may extend beyond vision needs. For example, older people's pain needs are often overlooked or dismissed (Clarke et al. 2014; Schofield et al. 2022) and this omission is likely to be significantly exacerbated for those with sensory issues (hearing and vision loss) particularly as pain assessment (often using visual assessment tools) is more challenging (Coker et al. 2010). Furthermore, older people with vision impairment are at higher risk for falls (Manners et al. 2024) and poorer mental health (Dale 2010). We would therefore strongly advocate for further training for healthcare staff and endorse the British Geriatrics Society (2023) proposal for holistic, multidisciplinary assessment, rehabilitative support, and follow up support and would add that this is particularly salient for those with recently acquired sight loss post discharge.

The environmental design of the hospital setting did not accommodate patients with vision impairment (objects out of reach, no guidance to use the bathroom or navigate the shower). The hospital ward was experienced as disabling, not only in terms of design and physical space, but also through process and temporality in which routines undermined independence and invalidated participants' sense of self, contributing again to a sense of invisibility and loss of identity. Being moved in the night, lack of orientation, removal of medications, and poorly designed equipment, all served to reduce adaptation, independence, and increase the risk of falls. The use of audio and digital media offered some sanctuary of the self and resistance to an undermining environment. Media offered both a practical space in which to communicate with family and assert identity and connection and also reflects the retreat into the safe space of a micro world. Such a retreat is also symbolic of a liminal space described by Turner (1969), (cited in Watermeyer 2013) as place of ‘between and betwixt’ reflecting the rendering of invisibility created by the care environment.

On a micro level in terms of daily caregiving, participants in our study valued care interactions which rendered them as visible. Caregiving described as going the ‘extra mile’ and which reflected patient centred care that were highly valued in counteracting care which in many ways, was experienced as neglect. We would advocate that as a minimum, practice should attend to equality, diversity, and inclusivity in promoting wellbeing as bound in the UK Equality Act GOV.UK (2010). We would also advocate revisiting models of person‐centred care, including dignity and compassion frameworks such as relationship centred care using a Senses Framework of belonging and significance (Nolan et al. 2004) in informing vision impaired aware care both within health care settings and within nursing and allied health care curricula.

Returning to the second aim of the study, this work provides a unique and original contribution to researching with people living with vision impairment in ways which are inclusive, accessible, and co‐productive. Our findings run counter to those of Duckett and Pratt (2001) who advocate for one‐to‐one interviews and focus groups when working with people with vision impairment. The offer of one‐to‐one recordings or support to create personal accounts using individual technologies was resisted with participants indicating that ‘they did not wish to go there alone’. Instead, prompts which stimulated reflection around emotional touchpoints as described in user centred design Bate and Robert (2007) were poignant in focussing reflections. Our experiences reflect the work of (Fisher, Craig, and Chamberlain 2019) which points to the challenges for people to talk about everyday life without tactile or object elicitation and captures the work of Beudaert (2020), which argues that visual symbols can stimulate an embodied imagination and a visual memory in those with vision impairment. Nevertheless, we recognise the potential for refining and extending creative ways to engage with inclusive co productive approaches to ensure effective outcomes. Primarily however, we would reiterate Duckett and Pratt (2007) approach that participative research should be underpinned by the principles of social justice. There is potential for a user‐based design approach in making health care spaces inclusive for vision impaired patients and we would reiterate the Royal National Institute for the Blind (2024) recommendations that older people with vision impairment are actively involved in the co‐production of services to ensure these are accessible and relevant to their needs.

Participative involvement is a negotiation of organisations, stakeholders, and practices and requires consideration around valuing, trust, and agency as well as equality, diversity and inclusive practices (Rutter et al. 2024). Our experiences suggest that participants were vulnerable to retraumatising. We would recommend working within the parameters of participants' own social spaces as well as ensuring support mechanisms (e.g., key workers or counsellors) are available and working in collaborative and small groups. It should be noted that many were keen to support student learning as a tangible and positive outcome of their trauma. The ‘top tip’ resources developed have been distributed to their service users by the SRSB and have been shared with the local hospital trust and will underpin further engagement work as an awareness raising art installation at a festival of learning across the city. We would argue that being involved in the production of clear outputs and further engagement further validates and values participant involvement as central to outcomes of the study.

In terms of education and the student experience, the involvement of students in the study provided not only an opportunity for students to engage in ‘real world’ research but also promoted interprofessional learning around the need for vision aware care. We were initially concerned that students would not be sufficiently engaged; however, their skills, rapport, and commitment (mostly working on placement days off) were humbling. Professional course requirements limited their involvement but given the RCN demand for a more research active profession (Royal College of Nursing 2024), we would welcome further reflection and flexibility involving students directly in research as part of placement learning, with a particular focus on the concept of ‘student as partner’ model as outlined by Neary (2012). Anecdotally, we are aware that the experience has raised awareness of vision impairment and students have acted as ‘champions’ among their peers. We would support the UK Vision Strategy (Royal National Institute for the Blind 2024) that further work is required to increase awareness among health care professionals to ensure quality care for those affected by vision impairment, particularly as need is predicted to increase significantly in the future.

Finally, we were mindful to manage expectations. We were delighted with the following comment from participants who attended the report presentation:

“Our thanks to the team for consulting with us—it has resulted in something very powerful that will hopefully make a big difference” but also aware that change can be challenging and slow

### 6.1 Strengths and Limitations

The study is significant in highlighting the importance of vision aware care to support the older patient population. Our work demonstrates the possibilities of working together with health care students and vision impaired people in a participatory and co productive model to enhance learning and create inclusive and tangible outputs. We recognise the need to further refine and extend participatory and co productive approaches which can creatively and inclusively support vision impaired people in the sharing their stories. Our older vision impaired participants were white and although there was diversity within in the student group, culturally and ethnically diverse insights of older people are absent. In many ways, this reflects current numbers in white to minority ethnic older people presenting with vision impairment (Royal National Institute for the Blind 2021). However, vision needs are projected to increase in ageing ethnic minority community groups (Heinze and Jones 2024) and the perspectives of older people from diverse backgrounds will be increasingly important to ensure that care and services can meet the needs of all. We are also aware that the study setting acted as a gatekeeper in the recruitment of participants and that potential sample bias may exist. Nevertheless, the charity provided onsite support which proved invaluable in supporting participant distress and the study would have been limited without the charity involvement.

The involvement of nursing, speech and language, and orthoptic students offered an opportunity for interprofessional learning and provided some minority ethnic perspective. However, preparation for research skills including facilitation, transcription, and analysis were assumed via undergraduate research teaching. Further work around preparation of students may be of value; however, from audio recordings, it was clear that students were highly skilled and empathetic in their interactions as facilitators and participants appeared to value the opportunity to support learning. Further embedding of impactful approaches to learning and practising research will, however, require flexibility within professional programmes.

### 6.2 Recommendations for Further Research

An audio‐visual learning resource based on the experiences of participants is being developed as part of a vision care aware learning package based. An evaluation of the resource will be invaluable in contributing to nursing and allied health professionals' curricula and learning. Second, we would also recommend a further focus using user centred participatory design approaches to explore hospital care environments, focussing on care practices and design and would aim to ensure an inclusive sampling strategy. Finally, further work is needed to refine inclusive and creative participatory and coproductive approaches to ensure effective and relevant outcomes for people with vision impairment.

### 6.3 Implications for Policy and Practice

Vision aware care is an increasing priority in the context of an ageing population with implications for physical and mental health wellbeing as well as ensuring equality diversity and inclusion (EDI) in practice. Involving pre‐registration nursing students in participatory and coproductive studies offers a powerful means of learning not only about research approaches but also the lived experience of care and care needs. Consideration is required to incorporate learning opportunities within the remit of professional programmes and requirements. Co‐creative approaches which engage in touch and tactility and support small group working can empower and support a user‐based design approach to working with vision impaired people.

## 7 Conclusions

Experiencing vision impairment is traumatic, however, the needs of older people with vision impairment can be overlooked in the acute hospital care setting. Supporting vision impaired older people through diagnosis, care, and enablement is vital for promoting wellbeing and quality of life but requires attention to issues of EDI and person‐centred care. Embedding of ‘real world’ research opportunities within professional programmes can support evidence‐based learning. Further work using user centred participative design approaches is needed to ensure that acute hospital care settings are inclusive, vision impaired friendly in design and care delivery, particularly in the context of an ageing population.

## Author Contributions

Gemma Arblaster, Fiona Wilson, Holly Geraghty, Sydney Graveling, Zahra Hussain, Nicola Jackson, Zaina Qamar, Elliot Rook and Elena Starsong made substantial contributions to conception and design, or acquisition of data, or analysis and interpretation of data. Fiona Wilson and Gemma Arblaster were responsible for drafting the manuscript or revising it critically for important intellectual content. Fiona Wilson and Gemma Arblaster provided final approval of the version to be published. Each author should have participated sufficiently in the work to take public responsibility for appropriate portions of the content. Fiona Wilson and Gemma Arblaster agreed to be accountable for all aspects of the work in ensuring that questions related to the accuracy or integrity of any part of the work are appropriately investigated and resolved.

## Conflicts of Interest

The authors declare no conflicts of interest.

## Peer Review

The peer review history for this article is available at https://www.webofscience.com/api/gateway/wos/peer‐review/10.1111/jan.16648.

## Data Availability

Data available on request from the authors.
